# Influence of Obstructive Jaundice on Pharmacodynamics of Rocuronium 

**DOI:** 10.1371/journal.pone.0078052

**Published:** 2013-10-16

**Authors:** Zhen-Meng Wang, Peng Zhang, Mi-Jia Lin, Bo Tan, Hai-Bo Qiu, Wei-Feng Yu

**Affiliations:** 1 Department of Anesthesia and Intensive Care, Eastern Hepatobiliary Surgery Hospital, Second Military Medical University, Shanghai, China; 2 Department of Clinical Diagnosis, Changhai Hospital, Second Military Medical University, Shanghai, China; 3 Department of Anesthesia, The First Affiliated Hospital of Fujian Medical University, Fuzhou, Fujian Province, China; 4 Analytic Center, Fudan University School of Pharmacy, Shanghai, China; University of Pisa, Italy

## Abstract

**Background:**

Anesthetics are variable in patients with obstructive jaundice. The minimum alveolar concentration awake of desflurane is reduced in patients with obstructive jaundice, while it has no effect on pharmacodynamics and pharmacokinetics of propofol. In this study, we investigated the influence of obstructive jaundice on the pharmacodynamics and blood concentration of rocuronium.

**Methods:**

Included in this study were 26 control patients and 27 patients with obstructive jaundice. Neuromuscular block of rocuronium was monitored by acceleromyography. Onset time, spontaneous recovery of the height of twitch first (T1) to 25% of the final T1 value (Duration 25%, Dur 25%), recovery index (RI), and spontaneous recovery of train-of-four (TOF) ratios to 70% were measured. The plasma rocuronium concentrations were determined by high performance liquid chromatography using berberine as an internal standard.

**Results:**

There was no significant difference in onset time between the two groups. The Dur 25%, the recovery index and the time of recovery of the TOF ratios to 70% were all prolonged in the obstructive jaundice group compared with the control group. The plasma concentration of rocuronium at 60, 90 and 120 min after bolus administration was significantly higher in the obstructive jaundice group.

**Conclusions:**

The neuromuscular blockade by rocuronium is prolonged in obstructive jaundice patients, and therefore precautions should be taken in case of postoperative residual neuromuscular block. The possible reason is impedance of rocuronium excretion due to biliary obstruction and increased plasma unbound rocuronium because of free bilirubin competing with it for albumin binding.

## Introduction

Obstructive jaundice (OJ) is a condition of bile and bile component retention due to extrahepatic or intrahepatic bile duct obstruction. Extrahepatic cholestasis means obstruction of large bile ducts outside the liver due to the formation of gallstones, cyst or malignant tumors [1]. Anesthetics are variable in OJ patients. It was found in our previous work that the minimum alveolar concentration awake of desflurane was reduced in OJ patients compared with nonjaundice controls [2], while other studies demonstrated obstructive jaundice had no effect on pharmacodynamics and pharmacokinetics of propofol [3,4].

Rocuronium bromide, an aminosteroid non-depolarizing neuromuscular blocking agent, is widely used for clinical anesthesia because of its short onset time and intermediate duration of action [5]. It’s reported that hepatic uptake and biliary excretion were the main mechanisms for its metabolism [6]. It is accepted that drug pharmacodynamics and pharmacokinetics undergo changes in hepatic and renal disease states [7-11]. However limited data are available on the influence of OJ on pharmacodynamics and the blood concentration of rocuronium. 

If pharmacodynamics and drug metabolism change in condition of obstructive jaundice, dosage adjustment or other measures may be recommended. Therefore, we hypothesized that the neuromuscular blockade by rocuronium was prolonged in OJ patients [12]. The aim of the present was to study the neuromuscular effect and blood concentration of rocuronium in OJ patients.

## Materials and Methods

### Patients

The study was approved by the Clinical Research Ethics Committee of the Eastern Hepatobiliary Surgery Hospital (Shanghai, China) and written informed consent was obtained from each patient scheduled for elective surgery because of bile duct disease. Thirty patients with OJ due to calculus or neoplasm in the bile duct and 30 control patients diagnosed as choledochal cyst, calculus or neoplasm in the bile duct (ASA class I or II) were studied. Exclusion criteria included age >65 yr or <18 yr; body mass index (BMI) >24.9 or <18.5; albumin <35 g/L; prothrombin time >14.0s; hepatic encephalopathy, ascites, acute cholangitis, or gastrointestinal hemorrhage; renal inadequacy; diabetes mellitus, electrolyte or acid-base disturbance; psychiatric illnesses, or neuropathy; sepsis; receiving drugs known to interfere with neuromuscular transmission [13].

### Anesthesia

All patients received intramuscular atropine 0.01 mg·kg^-1^ and morphine 0.15 mg·kg^-1^ 45 min before the induction of anesthesia. On arrival in the operating theatre, ECG, pulse oximetry and non-invasive arterial pressure monitoring were performed in all patients. Next, the right internal jugular vein or subclavian vein was cannulated. Epidural puncture was performed with the patients in a right lateral decubitus position at the T_8_-T_9_ intervertebral space using a paramedian approach. After identification of the epidural space by the loss of resistance to injection of air, an epidural catheter was threaded through the needle and inserted cephalad 4 cm.

General anesthesia was induced using fentanyl 2 μg·kg^-1^ and propofol 2 mg·kg^-1^ intravenously. After loss of consciousness, a laryngeal mask (SLIPA, Jiaxing Tongcheng Medical Appliance CO Ltd., Zhejiang, China) was inserted without the aid of neuromuscular blocking agents. Anesthesia was maintained with 100% oxygen and a propofol infusion at the rate of 8-12 mg·kg^-1^·h^-1^. When the T1 disappeared after rocuronium administration, all patients were given 5 ml 2% lidocaine epidurally as a test dose. If there was no complication in five min, the patients received a bolus of 10 ml 0.75% ropivacaine epidurally, followed by 5 ml 0.75% ropivacaine every 45-50 min. Ventilation was adjusted to achieve end-tidal carbon dioxide pressure of 30-35 mmHg using the Julian anesthesia machine (Drager Medical AG&Co. KG, Lubeck, Germany). The right radial artery was cannulated for invasive arterial blood monitoring and arterial blood gas analysis. The skin temperature over the thenar muscle was measured throughout the study period via the surface probe attached to the TOF Watch SX (Organon Ltd., Dublin, Ireland) and was maintained above 32°C by wrapping the arm in cotton wool and warming the infusion solution with a Ranger Blood/Fluid Warming system (Arizant Healthcare Inc., MN, USA).

### Neuromuscular monitoring

Once a stable depth of anesthesia was achieved, the forearm was well immobilized on a splint. Then the left ulnar nerve was stimulated at the wrist with square-wave pulse of 0.2 ms duration delivered at a frequency of 2 Hz in a TOF mode with 15 s intervals, and the force of contraction of the ipsilateral adductor pollicis muscle was measured using the TOF Watch SX for several times. TOF Watch SX was calibrated or recalibrated until the T1 twitch height deviated from 100% by less than 5%. After T1 responses had been stable for more than five min, 0.9mg/kg rocuronium (3×ED95) was administered i.v. within five seconds. All the rocuronium used in this study was stored at 4°C and had the same batch No. (471722). The neuromuscular monitoring was discontinued when TOFR reached 80%. 

The following variables were measured or calculated: onset time (sec) from initiation of the rocuronium injection to 95% depression of T1; duration 25% (Dur 25%), the time from initiation of rocuronium administration to spontaneous recovery of T1 height to 25% of the final T1 value; recovery index (min), spontaneous recovery of T1 from 25% to 75% of the final T1 value; TOFR70 (min), spontaneous recovery of TOF ratios to 70%. All data were collected and analyzed on a desktop computer with the TOF Watch SX monitor software (Version 2.2.INT). 

### Blood samples and rocuronium concentration analysis

Venous blood samples (3 ml) were taken 2 min prior to and at 30, 60, 90, 120 and 180 min following administration of rocuronium. The samples were anti-coagulated with sodium heparin and were immediately centrifuged at 2500 rpm for 5 min. Plasma (2 ml) was mixed with 1 M sodium dihydrogen phosphate (0.5 ml) and stored at -80°C for further analysis. 

A 1200 HPLC system (Agilent Technologies, CA, USA) consisting of a G1322A degasser, a G1311A quaternary pump, a G1329A well-plate autosampler and a G1316A thermostated column compartment was used. An API 4000 mass spectrometer (Applied Biosystems Sciex, MA, USA) equipped with a Turbo VTM ion source was connected to the LC system. Rocuronium and berberine standards were purchased from the National Institutes for Food and Drug Control, China. All solvents were of HPLC grades.

Rocuronium concentrations were measured by tandem mass spectrometry using berberine as an internal standard [14]. The separation was performed on an Agilent Zorbax SB-C18 column (150 mm×2.1 mm, 5μm) with a mobile phase of acetonitrile-ammonium acetate (10 mmol·L^-1^, 0.1% formic acid) (65:35) at a flow rate of 300 μl·min^-1^. The multiple reaction monitoring (MRM) mode with the transitions of m/z 529.2 → m/z 487.5 (rocuronium) and m/z 336.1 → m/z 292.1(IS) was used. The limits of detection were 5 ng·ml^-1^ and the linear calibration range was 0.030-12 μg·ml^-1^ (r = 0.9994) with an accuracy varying from 95.7% to 105%. The inter-batch precision (RSD%, n = 3) for plasma quality control samples varied from 1.0% to 1.7% of the nominal value. The intra-batch precision for three sets of plasma quality-control samples varied from 1.7% to 8.8% of the nominal value. 

### Statistical analysis

Statistical calculations were performed using the SPSS 13.0 software packages. The results are expressed as the mean ± SD; P values equal to or less than 0.05 were considered statistically significant.

## Results

### Patients

Three patients in the OJ group and four in the control group were excluded due to unstable neuromuscular transmission monitoring. Finally, 27 OJ patients and 26 control patients were included for analysis. The two groups were comparable for age, weight, height, body mass index (BMI), albumin and prothrombin time (PT). Total bilirubin, conjugated bilirubin and unconjugated bilirubin were significantly different between the two groups (Table 1). 

**Table 1:** The physical characteristics of patients in the two groups.

	*OJ (n=27)*	*Control (n=26)*
Age (year)	51.50 ± 10.26	49.07 ± 10.35
Weight (kg)	62.09 ± 8.21	56.38 ± 9.00
Height (m)	1.67 ± 0.08	1.62 ± 0.07
Body Mass Index (kg/m^2^)	22.11 ± 1.55	21.29 ± 1.82
Gender (M/F)	16/11	14/12
Albumin (g/L)	40.28 ± 2.98	41.54 ± 3.37
Prothrombin Time (s)	11.19 ± 0.90	11.19 ± 0.63
Urea (mmol/L)	4.84 ± 2.08	5.31 ± 1.82
Creatinine (µmol/L)	53.91 ± 9.72	56.23 ± 10.49
total bilirubin (µmol/L)	180.08 ± 75.42*	13.58 ± 3.91
conjugated bilirubin (µmol/L)	138.80 ± 60.39*	5.87 ± 2.12
unconjugated bilirubin (µmol/L)	42.68 ± 20.68*	7.77 ± 2.68

Data are shown as the means ± SD. **P<0.01 compared with the control group.

### Pharmacodynamics

There was no significant difference in onset time between two groups. The mean recovery time was prolonged in OJ group as compared with that in the control group. The prolongation of Dur 25% was statistically significant between the two groups (P<0.05). The recovery index and the time of the TOFR to 70% were both significantly different between the two groups (P<0.05, Table 2). 

**Table 2:** The pharmacodynamic variables of patients in the two groups.

	*OJ (n=27)*	*Control (n=26)*
Onset time (s)	62.05 ± 15.56	76.15 ± 31.50
Dur 25% (min)	80.78 ± 16.92*	62.83 ± 13.22
Recovery index (min)	31.86 ± 8.91**	20.63 ± 9.24
TOFR70 (min)	140.60 ± 29.40*	106.19 ± 23.64

Data are shown as the means ± SD. * *P*<0.05 compared with the control group. ** *P*<0.01 compared with the control group.

### Rocuronium blood concentration

**Figure 1:**
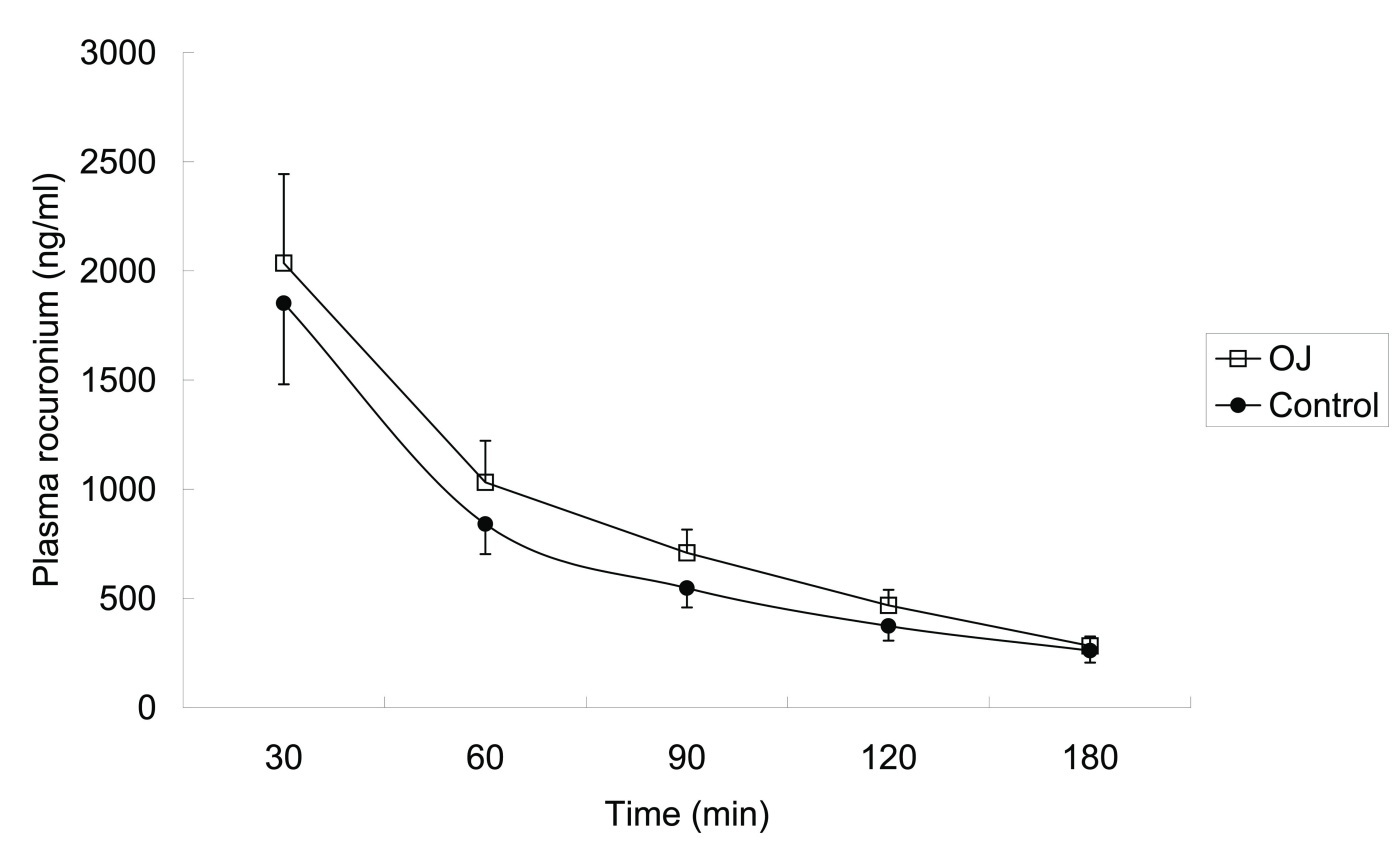
Mean plasma rocuronium concentration versus time for the two groups. Analysis reveals significant differences (*P*<0.05) at time of 60, 90 and 120 min between the two groups. (□ = obstructive jaundice group, n=11; ●=control group, n=10).

The plasma rocuronium concentrations were significantly higher in OJ group at 60, 90 and 120 min after bolus administration compared with those in control group. At 180 min after rocuronium administration, the concentration in OJ group was higher than that in control group (282.09±43.54 *vs* 260.8±55.05), though the difference was not statistically significant. The mean plasma rocuronium concentrations in the two groups are shown in Figure 1. 

## Discussion

In the present study, we found that the neuromuscular block of rocuronium was prolonged in OJ patients as compared with control patients. Dur 25%, recovery index and recovery of TOF ratio to 70% were all significantly prolonged in OJ group. The blood concentration of rocuronium at 60, 90 and 120 min after bolus administration was significantly higher in OJ patients.

Suzuki et al [15] demonstrated that the time of spontaneous and facilitated recovery to a TOF ratio of 75% and 90% was significantly delayed by epidurally administration of mepivacaine without affecting T1 recovery and recovery index. In the present study, we combined general anesthesia with epidural anesthesia, and found that it provide satisfactory muscular relaxation for operation, thus making it possible to monitor the entire recovery after bolus rocuronium administration. During recovery, the final T1 heights of the two groups of patients were 80-100% of the baseline values, indicating that the T1 height was not affected by epidural injection ropivacaine. In our study, recovery parameters related with T1 height were adjusted to the final T1 height, which made the results accurate and reliable.

Rocuronium, a monoquaternary aminosteroidal non-depolarizing neuromuscular blocking agents, was first available in 1994. It has a similar pharmacodynamic and pharmacokinetic profile with vecuronium, except for it has a more rapid onset of action and a longer duration. Rocuronium at a dose of 0.6 mg/kg can provide a clinically acceptable intubation condition in 60-90 seconds in the majority of children, adults and elderly patients [16]. After spontaneous recovery, the mean clinical duration, mean recovery index and mean recovery of TOFR to 0.7 of rocuronium (0.6 mg/kg, 2×ED_95_) were 24-43, 9-14, and 47-72 minutes, respectively [17].

Animal studies have suggested that rocuronium is cleared by the hepatic uptake and biliary excretion in the unchanged drug; in cats, only 8.7% of a bolus dose was detected in the urine over 24 h [18]. Therefore the neuromuscular blockade of an equipotent single dose of rocuronium may be prolonged and complicated in patients with liver and kidney inadequacy. In patients with hepatic cirrhosis, the mean recovery time and the time to recovery of the TOF ratio to 70% of rocuronium were prolonged due to the reduced plasma clearance, as compared with the healthy group. It was demonstrated that the clinical duration and the time of recovery of the TOF ratio to 70% were significantly prolonged in the renal failure group compared to those in the group with normal renal function. Robertson et al [19] concluded that this increase might be due to a decreased clearance of rocuronium. Kocabas et al [6] found the time to recovery of the T1 to 25%, 50%, 75% and 90% of the control value and TOF ratio to 70% and recovery index were prolonged in both young and elderly patients with renal failure as compared with healthy control. 

It was found in this study that the conjugated bilirubin was significantly elevated in OJ group with calculus or neoplasm in the bile duct, indicating that the jaundice was caused by obstruction of bile duct. The albumin, prothrombin time, urea and creatinine were in the normal range and neither group demonstrated any ascites or hepatic encephalopaghy, indicating that none of the patients in the two groups had renal or hepatic dysfunction. It could therefore be concluded that the prolonged neuromuscular effect of rocuronium in OJ patients was caused by some other mechanisms. 

The biliary excretion of cefpiramide [8], a non-metabolized, highly-biliary excreted antibiotic, was markedly diminished in rats with experimentally-induced OJ. Therefore, we measured the plasma rocuronium concentration by tandem mass spectrometry using berberine as an internal standard. The plasma concentration of rocuronium at 60, 90 and 120 min after bolus administration was significantly higher in OJ patients. 

The present study also found that the neuromuscular effect of rocuronium was prolonged and the blood concentration of rocuronium at 60, 90 and 120 min after bolus administration was significantly higher in OJ patients. Based on the finding of the present study and previous reports, we suggest that prolongation of neuromuscular blockade in OJ patients receiving rocuronium maybe due to its impeded excretion caused by bile duct obstruction. There may be another possibility for the prolonged neuromuscular blockade of rocuronium. It was reported [20] that serum free bilirubin competed with drugs for albumin binding, thus affecting the unbound drug concentrations. Knowing that the albumin binding rate of rocuronium is about 25% [21], the increased free bilirubin in our study might compete with rocuronium for albumin binding, resulting in a higher fraction of unbound rocuronium. These two mechanisms may underlie the prolonged neuromuscular blockade of rocuronium in OJ patients. 

In conclusion, the neuromuscular blockade of rocuronium was prolonged in OJ patients, and the possible reason is the impeded excretion of rocuronium due to bile duct obstruction and/or increased concentrations of plasma unbound rocuronium due to free bilirubin competing with rocuronium for albumin binding. Objective neuromuscular monitoring, sugammadex [22] or anti-cholinesterase [23] is recommended for OJ patients to reduce the incidence of pulmonary complications and post-operative residual neuromuscular blockade.
